# In Vitro Killing Activities of Anidulafungin and Micafungin with and without Nikkomycin Z against Four *Candida auris* Clades

**DOI:** 10.3390/pharmaceutics15051365

**Published:** 2023-04-29

**Authors:** Awid Adnan, Andrew M. Borman, Zoltán Tóth, Lajos Forgács, Renátó Kovács, Dávid Balázsi, Bence Balázs, Gergely Udvarhelyi, Gábor Kardos, László Majoros

**Affiliations:** 1 Department of Medical Microbiology, Faculty of Medicine, University of Debrecen, 4032 Debrecen, Hungary; 2 Doctoral School of Pharmaceutical Sciences, University of Debrecen, 4032 Debrecen, Hungary; 3 UK National Mycology Reference Laboratory, UK Health Security Agency, Science Quarter, Southmead Hospital, Bristol BS10 5NB, UK; 4 Medical Research Council Centre for Medical Mycology (MRC CMM), University of Exeter, Exeter EX4 4QD, UK; 5 Department of Metagenomics, University of Debrecen, 4032 Debrecen, Hungary

**Keywords:** time–kill methodology, synergism, echinocandins, chitin synthase inhibitor

## Abstract

*Candida auris* is a multidrug-resistant pathogen against which echinocandins are the drug of choice. However, information on how the chitin synthase inhibitor nikkomycin Z influences the killing activities of echinocandins against *C. auris* is currently lacking. We determined the killing activities of anidulafungin and micafungin (0.25, 1, 8, 16 and 32 mg/L each) with and without nikkomycin Z (8 mg/L) against 15 isolates representing four *C. auris* clades (South Asian n = 5; East Asian n = 3; South African n = 3; South American n = 4, two of which were of environmental origin). Two and one isolates from the South Asian clade harbored mutations in the hot-spot 1 (S639Y and S639P) and 2 (R1354H) regions of the *FKS1* gene, respectively. The anidulafungin, micafungin and nikkomycin Z MIC ranges were 0.015-4, 0.03-4 and 2->16 mg/L, respectively. Anidulafungin and micafungin alone exerted weak fungistatic activity against wild-type isolates and the isolate with a mutation in the hot-spot 2 region of *FKS1* but was ineffective against the isolates with a mutation in the hot-spot 1 region. The nikkomycin Z killing curves were always similar to their respective controls. Twenty-two of sixty (36.7%) anidulafungin plus nikkomycin Z and twenty-four of sixty (40%) micafungin plus nikkomycin Z combinations produced at least 100-fold decreases in the CFUs (synergy), with a 41.7% and 20% fungicidal effect, respectively, against wild-type isolates. Antagonism was never observed. Similar results were found with the isolate with a mutation in hot-spot 2 of *FKS1*, but the combinations were ineffective against the two isolates with prominent mutations in hot-spot 1 of *FKS1*. The simultaneous inhibition of β-1,3 glucan and chitin synthases in wild-type *C. auris* isolates produced significantly greater killing rates than either drug alone. Further studies are warranted to verify the clinical efficacy of echinocandin plus nikkomycin Z combinations against echinocandin susceptible *C. auris* isolates.

## 1. Introduction

According to the World Health Organization, *Candida auris* is a critical priority fungal pathogen [1]. This classification underlines the need for novel and highly effective therapies against this emerging microorganism, which is associated with hospital outbreaks and the rapid emergence of resistance against conventional antifungal agents [2,3,4,5,6]. Recent guidelines recommend echinocandins as the drug of choice for severe *C. auris* infections [2,3,4,7,8]. However, clade-specific mortality is still high, especially among patients with COVID-19 infection, where 66%, 60% and 50% mortality rates were reported with the South American, South Asian, and South African clades, respectively [9,10,11]. Moreover, reports of echinocandin-resistant isolates emerging during treatment are also increasing [1,2,4]. Although antimicrobial peptides, metals, nanoparticles and natural compounds (i.e., quorum-sensing molecules) are often promising new approaches, their translation to clinical practice is often delayed due to the lack of safety and efficacy data in mammals [12,13,14,15]. To address the low activity of antifungal agents, numerous combinational therapies have been proposed, based mostly on in vitro data. Suggested combinations include multiple antifungal agents together, antifungal agents with antibiotics and antifungal agents combined with nonantimicrobial drugs [12,13,14,15].

Although nikkomycin Z is an obvious choice for this purpose, as phase I clinical results that support its tolerability in humans up to 2000 mg single per os dose are available [16,17,18], data on its activity against *C. auris* are scarce. Nikkomycin Z is a peptidyl nucleoside derived from *Streptomyces tendae*, with a novel mechanism of action targeting chitin synthesis of the fungal cell wall. It shows considerable activity against hard-to-treat fungi, such as *Blastomyces* and *Coccidioides* [18]. While the activity of nikkomycin Z against common *Candida* species alone is mediocre at best, synergism was commonly observed in combination with echinocandins, resulting in faster killing [18,19]. In animal models, nikkomycin Z was well tolerated. However, after intravenous and oral administration only a short drug half-life (15 and 60 min, respectively) was detected. Development is limited only by financial issues related to large-scale production and commercialization [18].

The aim of our study was to investigate the interaction of nikkomycin Z with micafungin and anidulafungin using a time–kill methodology and to assess whether this combination can overcome the echinocandin tolerance of *C. auris* isolates belonging to various lineages.

## 2. Materials and Methods

### 2.1. Isolates

Fifteen clinical isolates representing four of the five described *C. auris* clades (South Asian n = 5; East Asian n = 3; South African n = 3; South American n = 4) were used in this study. Two isolates from the South American clade were derived from the hospital environment. The type strain (NCPF 13029 = CBS 10913; East Asian clade) was also included (Table 1). *C. auris* isolates were identified by a combination of ribosomal DNA gene sequencing targeting the 28S rRNA and/or ITS1 regions, which was also used for clade delineation [20,21,22]. Two days before the experiments, the isolates were subcultured using Sabouraud agar and screened on CHROMagar *Candida* (Becton Dickinson) to ensure the purity of the *Candida* isolates. 

### 2.2. Whole Genome Sequencing

Library preparation was performed using the tagmentation-based Illumina DNAFlex Library Prep kit (Illumina, San Diego, CA, USA), according to the manufacturer’s protocol. Paired-end 300 bp sequencing was executed on an Illumina MiSeq instrument. The raw sequencing reads were aligned to the *C. auris* B8441 reference genome using the Burrows–Wheeler Aligner algorithm. The genetic variants (single-nucleotide polymorphisms, mutations and indel variants) were determined using the GATK algorithm. The library preparations, sequencing and data analysis were performed at the Genomic Medicine and Bioinformatics Core Facility of the University of Debrecen, Hungary [23]. Two isolates (28 and 208) from the South Asian clade harbored mutations (S639Y and S639P, respectively) in hot-spot 1 of the *FKS1* gene region, as determined by whole genome sequencing. In the case of isolate 20 (South Asian clade), a mutation (R1354H) was found in hot-spot 2 of the *FKS1* gene. The remaining 12 isolates showed wild-type genotypes (Table 1).

### 2.3. Antifungal Susceptibility Testing

We conducted the MIC assays in U-bottom, tissue culture-treated microtiter test plates (TPP Techno Plastic Products AG, Switzerland; cat. no. 92097). Anidulafungin and micafungin were obtained from Molcan Corporation (Richmond Hill, ON, Canada). Nikkomycin Z was purchased from Sigma (Budapest, Hungary). Antifungals were dissolved in 100% DMSO and diluted further in RPMI 1640 to final concentrations between 0.015 and 8 mg/L for anidulafungin or micafungin and 0.03 and 16 mg/L for nikkomycin Z. The starting inoculum was ~10^3^ CFU/mL. Plates were incubated at 35 °C, and MICs were read visually after 24 h using the partial inhibition criterion [24,25]. The MICs were determined at least in duplicate. For categorization, tentative MIC breakpoints, as suggested by the Centers for Disease Control and Prevention, were used: susceptible ≤ 2 mg/L for both anidulafungin and micafungin [7]. Clinical breakpoints or epidemiological cutoff values have not been published for nikkomycin Z against *Candida* species.

### 2.4. Time–Kill Studies

The anidulafungin and micafungin killing curves were determined with all isolates in RPMI 1640 at 0.25, 1, 8, 16 and 32 mg/L [21]. As previous pharmacokinetic studies have shown that nikkomycin Z concentrations in serum did not exceed 8 mg/L, the highest nikkomycin Z concentration tested was 8 mg/L [17,18]. Independently of measured nikkomycin Z MICs, the highest concentration of echinocandins tested were 32 mg/L for all isolates. The starting inocula were 2–6 × 10^5^ CFU/mL. The samples were removed at 0, 4, 8, 12 and 24 h, serially diluted ten-fold, plated (4 × 30 mL) onto a single Sabouraud dextrose agar and incubated at 35 °C for 48 h. The limit of detection was 50 CFU/mL. Fungicidal or fungistatic activity was defined as ≥3 log CFU/mL or <3 log CFU/mL changes in the viable cell count compared to the starting inoculum. All experiments were performed twice; the means of the resulting data are presented. The killing kinetics at the tested concentrations were analyzed, as described previously [21]. Positive killing rate (*k*) values indicate killing, and negative *k* values indicate growth. The mean times to achieve a 99.9% reduction of the starting inoculum (T_99.9_ = 3/*k*) were calculated from the *k* values for each isolate and concentration [21].

### 2.5. Combination of Anidulafungin and Micafungin plus Nikkomycin Z in Time–Kill Studies

Nikkomycin Z was added at a single, fixed (8 mg/L) concentration to 0.25, 1, 8, 16 and 32 mg/L of anidulafungin and micafungin [19]. The efficacy was determined as described above, and the T_99.9_ values were calculated as well. Synergy and antagonism were defined, respectively, as a 100-fold increase or decrease in killing compared with the killing of the most active single antifungal. If the change was less than 100-fold, the interaction was considered additive. Interactions were determined after 24 h [22].

### 2.6. Statistical Analysis

One-way ANOVA with Tukey’s post-testing was used to analyze differences in the killing kinetics among isolates and concentrations of anidulafungin and micafungin with or without nikkomycin Z. The *t*-test (with Welch’s correction, where appropriate) was used for the same anidulafungin and micafungin concentrations to test differences in the killing kinetics of anidulafungin plus nikkomycin Z and micafungin plus nikkomycin Z [19,21].

## 3. Results

### 3.1. Anidulafungin, Micafungin and Nikkomycin Z MIC Values against four Candida auris Clades

The anidulafungin and micafungin MICs (with the exception of isolate 28, South Asian clade, with a mutation in hot-spot 1 of the *FKS1* gene) were not higher than the tentative MIC breakpoints suggested by the CDC [7]. The micafungin MICs were the same or 1–3 dilutions higher than the anidulafungin MICs (Table 1).

The nikkomycin Z MICs ranged from 2 to >16 mg/L; 5 of 15 of the isolates were not inhibited, even at 16 mg/L (Table 1).

#### 3.1.1. Nikkomycin Z Killing Activity against four *Candida auris* Clades

Regardless of the isolate and clade, nikkomycin Z did not produce even transient CFU decreases at the tested concentrations; the largest decrease measured was 0.17 log CFU in the case of isolate 20 (MIC = 2 mg/L, South Asian clade). The time–kill curves for isolates 20 and I-156 (MIC = 4 mg/L) (South Asian and South American clades, respectively) are shown in Supplementary Materials Figure S1.

#### 3.1.2. Anidulafungin and Anidulafungin plus Nikkomycin Z Killing Activities against Four *Candida auris* Clades

##### South Asian Clade

**Figure 1:**
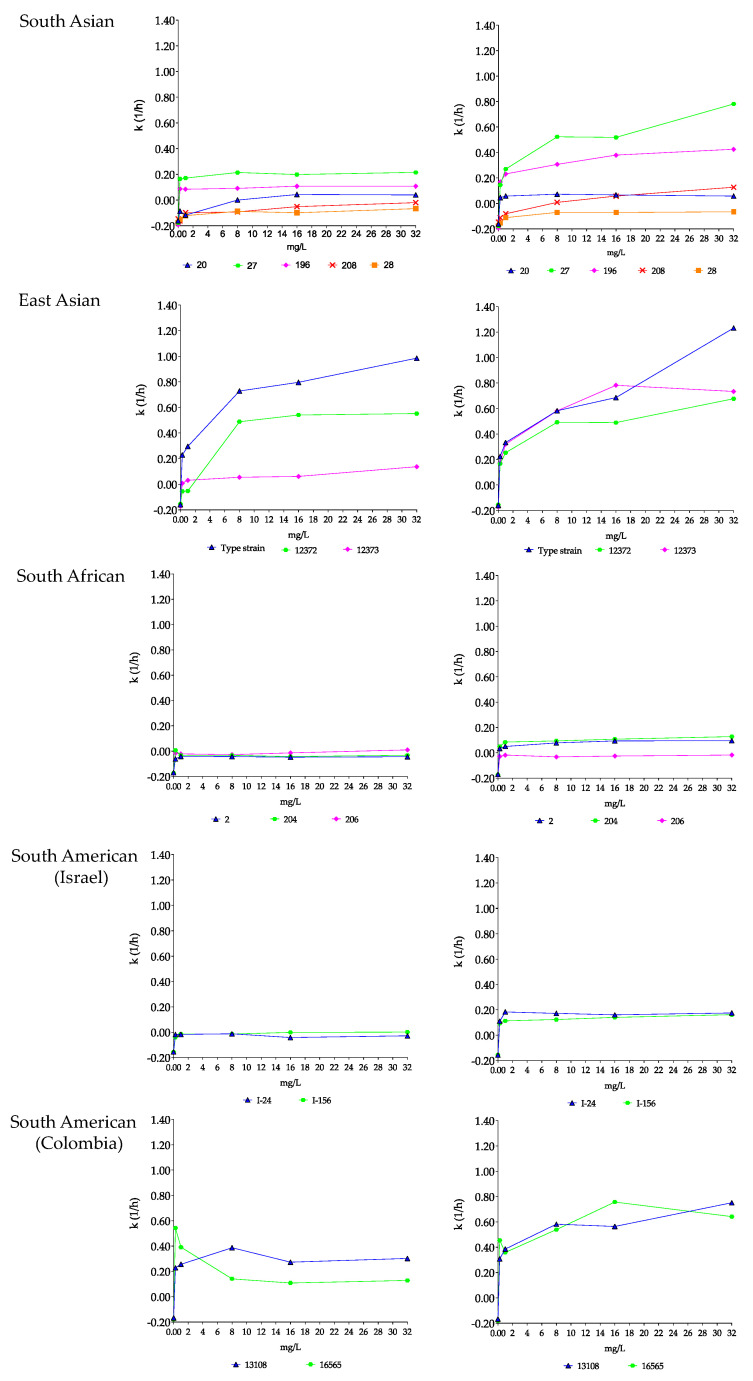
Killing rate (*k*) values of anidulafungin (**left**) and anidulafungin plus nikkomycin Z (**right**) in RPMI 1640 against South Asian (isolates 20, 27, 28, 196 and 208), East Asian (type strain = NCPF 13029 = CBS 10913, and isolates 12372 and 12373), South African (isolates 2, 204 and 206) and South American (isolates I-24 and I-156 from Israel and hospital environmental isolates 13108 and 16565 from Colombia) clades. Anidulafungin was tested at 0.25, 1, 8, 16 and 32 mg/L with (8 mg/L) and without nikkomycin Z. Positive and negative *k* values indicate a decrease and increase, respectively, in viable cell numbers. Error bars were omitted for better visualization of the graphics.

Anidulafungin showed fungistatic effect against the two wild-type isolates, and the highest CFU decreases (1.4 log) and *k* values (0.22 1/h) were found with isolate 27 at 8 and 32 mg/L (Table 2 and Figure 1). In the case of isolate 20, with a mutation in hot-spot 2 of FKS1, the *k* values were negative at 0.25 and 1 mg/L (−0.09 and −0.12 1/h, respectively). For the two isolates (28 and 208) with prominent mutations in hot-spot 1 of FKS1, anidulafungin produced a weak fungistatic effect in the first 4–8 h, with prominent regrowth and *k* values that were always negative after 24 h (Table 2, Figure 1 and Supplementary Materials Figure S2). The addition of 8 mg/L of nikkomycin Z to anidulafungin increased the *k* values for the two wild-type isolates (Table 2 and Figure 1). With isolate 196, synergy (more than 2 log CFU decreases) was noted with a fungicidal effect from 1 to 32 mg/L anidulafungin plus 8 mg/L nikkomycin Z (T_99.9_ ranged from 4.7 to 8.7 h) at all tested concentrations (Table 2). Although the nikkomycin Z MICs for isolates 20 and 27 were higher than 16 mg/L, 8–32 mg/L anidulafungin plus 8 mg/L nikkomycin Z was fungicidal in the case of isolate 27 (T_99.9_ ranged from 2.6 to 5.8 h, Supplementary Materials Table S1), and the *k* values were positive at all tested combinations (*k* value ranges were 0.05–0.07 1/h) in the case of isolate 20. However, synergy was not observed for these isolates.

In the case of isolate 28, the addition of nikkomycin Z to anidulafungin produced only transient CFU decreases after 4 h, followed by marked regrowth after 24 h (0.54–1.42 log CFU/mL increases; *k* values ranged from −0.07 to −0.15 1/h) (Table 2 and Figure 1). In contrast, in the case of isolate 208, 8–32 mg/L anidulafungin plus 8 mg/L nikkomycin Z produced positive *k* values; the highest *k* value (0.13 1/h) was found with 32 mg/L anidulafungin plus 8 mg/L nikkomycin Z (Table 2, Figure 1 and Supplementary Materials Figure S2).

##### East Asian Clade

**Figure 2:**
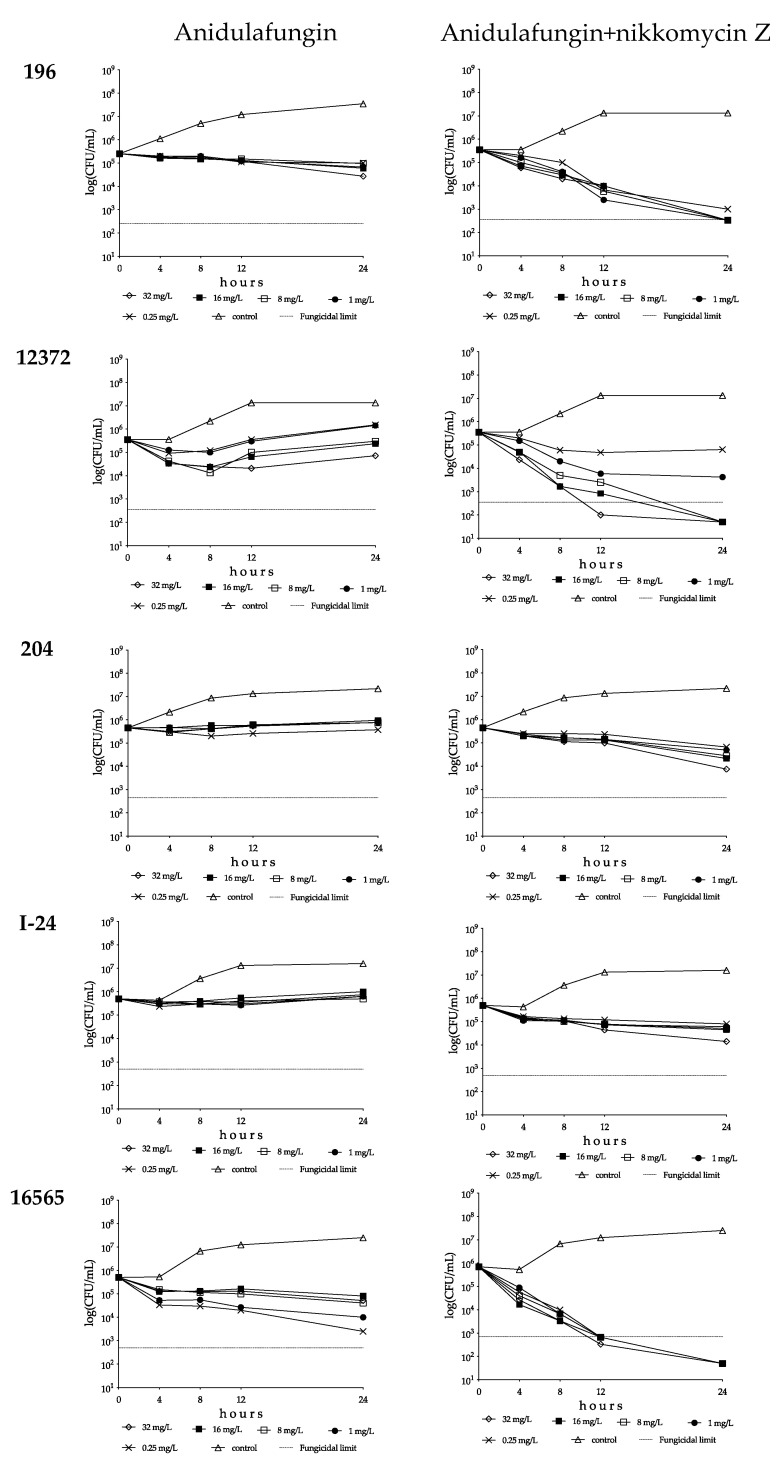
Time–kill plots of anidulafungin (**left**) and anidulafungin plus nikkomycin Z (**right**) in RPMI 1640 against *Candida auris* isolates 196 (South Asian clade), 12372 (East Asian clade), 204 (South African clade), I-24 (bloodstream isolate from Israel, South American clade) and 16565 (hospital environmental isolate from Colombia, South American clade). Anidulafungin was tested at 0.25, 1, 8, 16 and 32 mg/L with (8 mg/L) and without nikkomycin Z.

Anidulafungin showed concentration-dependent killing activity against the type strain with a 3 log CFU decrease at 32 mg/L (T_99.9_ was 3.04 h, Supplementary Materials Table S1). Against the remaining two isolates, anidulafungin was fungistatic and regrowth was always observed (Table 2 and Figure 1). In combination experiments, 16–32 mg/L anidulafungin with nikkomycin Z produced a rapid fungicidal effect (T_99.9_ = 1.7−2.9 h) against the type strain (Figure 1 and Supplementary Materials Table S1). Nikkomycin Z increased the killing activity of anidulafungin at all concentrations against isolates 12372 and 12373; 1–32 mg/L anidulafungin plus 8 mg/L nikkomycin Z proved to be synergistic against both isolates (Table 2). Moreover, 8–32 mg/L anidulafungin plus 8 mg/L nikkomycin Z produced a fungicidal effect (Figure 1 and Figure 2, Supplementary Materials Table S1).

##### South African Clade

Anidulafungin against the South African clade produced a weak fungistatic effect with *k* values that were almost always negative (Figure 1 and Figure 2, Table 2). A total of 8 mg/L nikkomycin Z increased the killing activity of anidulafungin against isolates 2 and 204 (*k* values were positive at all tested combinations; Figure 1 and Figure 2, Table 2), with synergy in the case of isolate 204 at 32 mg/L anidulafungin plus 8 mg/L nikkomycin Z (Table 2). Transient CFU decreases were found in the case of isolate 206 in the first 4–8 h, but after 24 h regrowth (0.08–0.20 CFU increases) was observed at the tested combinations (Figure 1 and Table 2).

##### South American Clade

Anidulafungin showed a negligible fungistatic effect against the isolates from Israel, with regrowth always observed (CFU increase ranges were 0.10–0.30 and 0.16–0.56 for isolates I-24 and I-156, respectively) (Figure 1 and Figure 2, Table 2). Nikkomycin Z significantly increased the killing activity of anidulafungin, with *k* values that were always positive at all tested combinations for both isolates (Figure 1 and Figure 2). In the case of isolate I-156, synergy was observed when 32 mg/L anidulafungin was combined with 8 mg/L nikkomycin Z (Table 2).

Anidulafungin was fungistatic against the environmental isolates of the South American clade, with higher *k* values at lower concentrations in the case of isolate 16565 (mini-paradoxical effect) (Figure 1 and Table 2). The combination of anidulafungin with nikkomycin Z produced a rapid fungicidal effect (T_99.9_ value ranges for isolates 13108 and 16565 were 1.1–6.6 and 2.6–5.6 h, respectively) (Figure 1 and Figure 2, Supplementary Materials Table S1). Moreover, 9 of the 10 drug combinations were synergistic (Table 2).

#### 3.1.3. Micafungin and Micafungin plus Nikkomycin Z Killing Activities against Four *Candida auris* Clades

##### South Asian Clade

**Figure 3:**
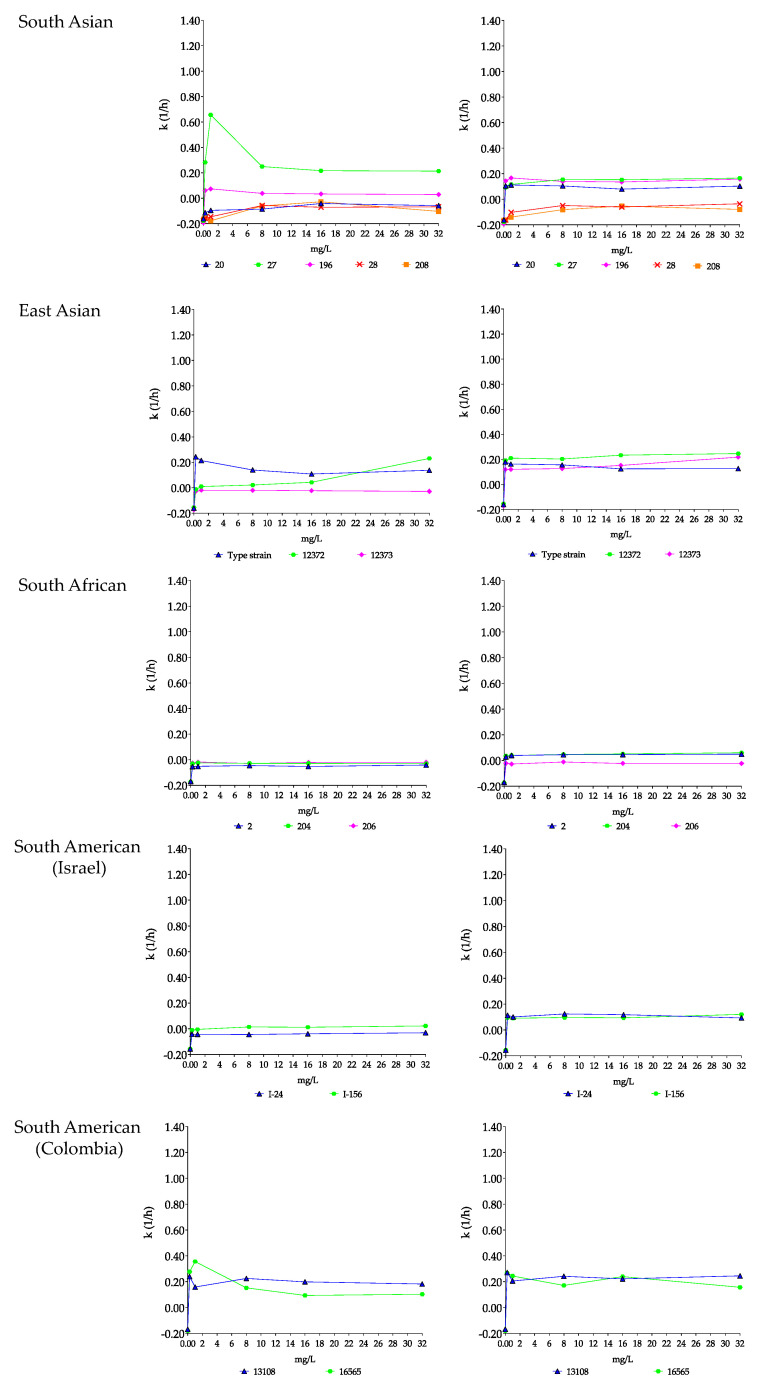
Killing rate (*k*) values of micafungin (**left**) and micafungin plus nikkomycin Z (**right**) in RPMI 1640 against South Asian (isolates 20, 27, 28, 196 and 208), East Asian (type strain = NCPF 13029 = CBS 10913, and isolates 12372 and 12373), South African (isolates 2, 204 and 206) and South American (isolates I-24 and I-156 from Israel and hospital environmental isolates 13108 and 16565 from Colombia) clades. Micafungin was tested at 0.25, 1, 8, 16 and 32 mg/L with (8 mg/L) and without nikkomycin Z. Positive and negative *k* values indicate a decrease and increase, respectively, in viable cell numbers. Error bars were omitted for better visualization of the graphics.

**Figure 4:**
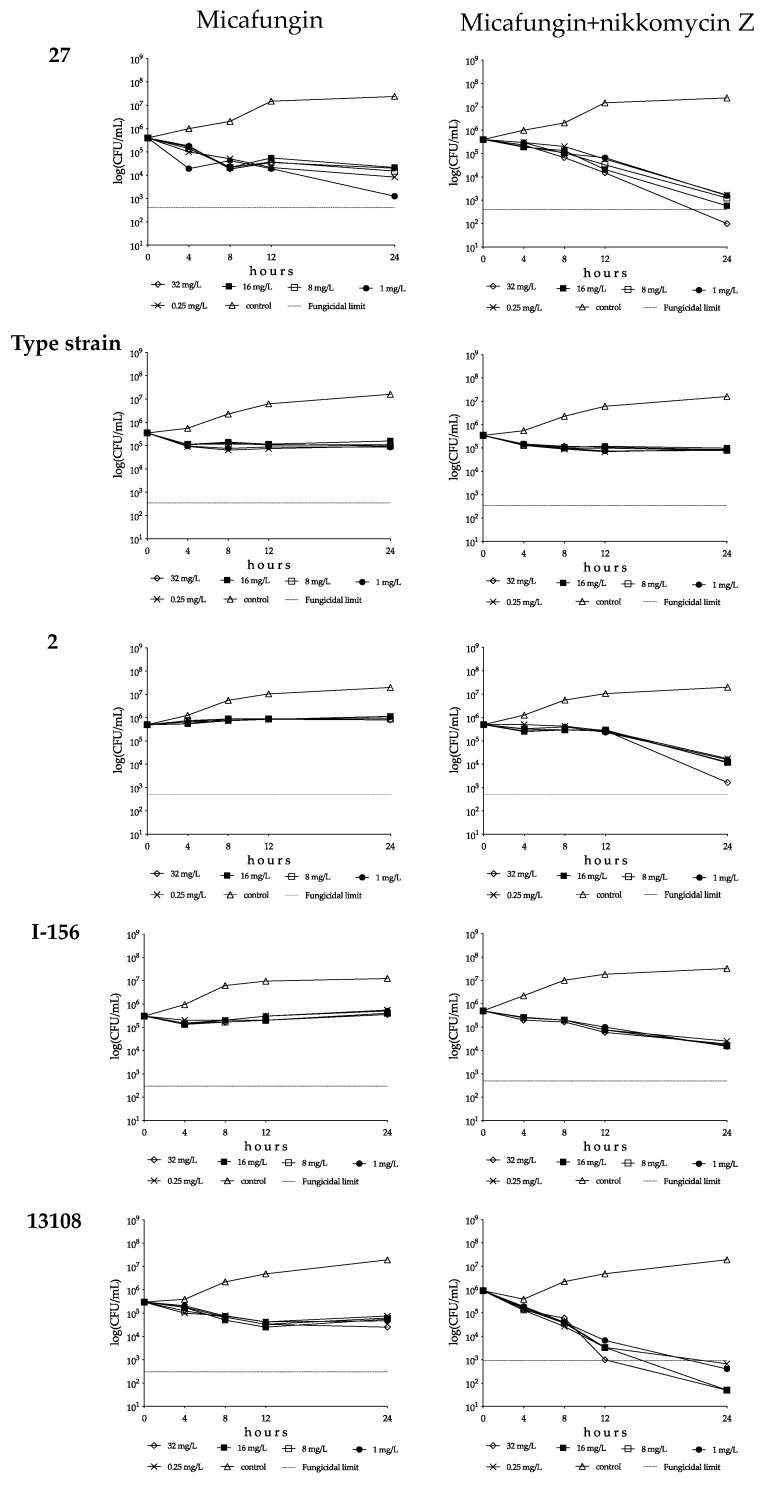
Time–kill plots of micafungin (**left**) and micafungin plus nikkomycin Z (**right**) in RPMI 1640 against isolates 27 (South Asian clade), NCPF 13029 = CBS 10913 (type strain, East Asian clade), 2 (South African clade), I-156 (bloodstream isolate from Israel, South American clade) and 13108 (hospital environmental isolate from Colombia, South American clade). Micafungin was tested at 0.25, 1, 8, 16 and 32 mg/L with (8 mg/L) and without nikkomycin Z.

With isolates 27 and 196, the CFU decreases were higher at 0.25 or 1 mg/L than at 8–32 mg/L (mini-paradoxical effect); the highest *k* value was measured with isolate 27 at 1 mg/L (k = 0.66 1/h) (Figure 3 and Figure 4, and Table 3). Micafungin produced small CFU decreases with prominent regrowth (0.32–0.85 log increases) and negative *k* values at 1–32 mg/L against isolate 20 (R1354H mutation in hot-spot 2 of FKS1). The killing activity of micafungin against this isolate increased significantly in the presence of 8 mg/L nikkomycin Z at all concentrations (*k* value ranges were 0.08–0.11 1/h), with synergy at 0.25–1 mg/L micafungin plus 8 mg/L nikkomycin Z (Figure 3 and Table 3). For isolates 27 and 196, four of ten drug combinations showed synergy (Table 3). Moreover, in the case of isolate 27, a slow fungicidal effect (T_99.9_ = 18.1 h) was observed at 32 mg/L micafungin plus 8 mg/L nikkomycin Z (Figure 4, Table 3 and Supplementary Materials Table S2). In contrast, against isolates 28 and 208 (mutations in hot-spot 1 of FKS1), micafungin with or without nikkomycin Z produced only transient CFU decreases in the first 4 h of exposure with prominent regrowth and negative *k* values after 24 h (Figure 3 and Table 3).

##### East Asian Clade

Micafungin was fungistatic against isolates from the East Asian clade, with regrowth almost always observed (Table 3). In the case of the type strain, the *k* values at 0.25–1 mg/L were significantly higher than at 8–32 mg/L (0.21–24 1/h and 0.11–0.14 1/h, respectively, Figure 3 and Figure 4). For isolate 12373, the *k* values were negative at all tested concentrations (Figure 3). In the combination experiments, for the type strain nikkomycin Z did not significantly alter killing (mini-paradoxical effect) compared to micafungin alone (Figure 3 and Figure 4). In contrast, for isolates 12372 and 12373, 8 of 10 drug combinations led to synergy, and a slow fungicidal effect (T_99.9_ = 23.7 h) was noticed with isolate 12373 at 32 mg/L micafungin plus 8 mg/L nikkomycin Z (Table 3 and Supplementary Materials Table S2).

##### South African Clade

Micafungin showed a weak fungistatic effect against the three isolates from this clade, with growth or transient CFU decreases (*k* values were always negative for the three isolates) (Figure 3 and Figure 4, and Table 3). Nikkomycin Z significantly increased the killing activity of micafungin at all tested concentrations with isolates 2 and 204. Moreover, in the case of isolate 2, synergy was noticed at the two highest micafungin concentrations (Figure 3 and Figure 4, and Table 3). In the case of isolate 206, similar to anidulafungin plus nikkomycin Z combinations, the *k* values remained negative at all tested combinations (*k* value ranges were from −0.01 to −0.03 1/h) (Figure 3).

##### South American Clade

Micafungin generated a weak fungistatic effect against clinical isolates; the *k* values were positive only in the case of isolate I-156 at 8–32 mg/L (0.01–0.02 1/h) (Figure 3 and Figure 4). The micafungin *k* values significantly increased with nikkomycin Z; at all tested concentrations micafungin plus nikkomycin Z produced concentration-independent killing activities (*k* value ranges were 0.09–0.12 1/h for both isolates) without a synergistic effect (Figure 3 and Figure 4, and Table 3).

Micafungin against the environmental isolates was fungistatic, and the *k* values were lower at 8–32 mg/L than at 0.25 or 1 mg/L (mini-paradoxical effect) (Figure 3 and Figure 4, and Table 3). Adding nikkomycin Z to micafungin produced a fungicidal effect with synergy in all combinations (10 of 10) for both isolates (Figure 3 and Figure 4, Table 3 and Supplementary Materials Table S2).

## 4. Discussion

The anidulafungin and micafungin MICs were lower than the tentative breakpoint (≤2 mg/L) suggested by the CDC for all tested wild-type isolates and two of three isolates with *FKS* mutations, a finding which may suggest the need to re-examine the breakpoints. With the exception of anidulafungin against the type strain (NCPF 13029 = CBS 10913) at 32 mg/L (Table 2), both tested echinocandins showed fungistatic activities against wild-type *C. auris* clinical isolates and the isolate with a mutation in hot-spot 2 of *FKS1* with frequent regrowth or, to a lesser extent, decreased killing activities at higher drug concentrations (mini-paradoxical effect). The two echinocandins were ineffective against the isolates with the known resistance mutation in hot-spot 1 of *FKS1*. Nikkomycin Z alone was not fungistatic against *C. auris* isolates, even at high drug concentrations (8–16× MIC values; Figure 1); the killing curves were always similar to their respective controls, even against isolates with lower MICs (2–4 mg/L). The killing activities of anidulafungin and micafungin against the wild-type isolates were significantly increased in the presence of nikkomycin Z with the exception of isolate 206 (South African clade). The extent varied considerably in an isolate-dependent manner, ranging from a slight increase in the maximum CFU decrease to a weak fungistatic effect turning to rapid fungicidality. The mini-paradoxical effect was also eliminated with nikkomycin Z, except in the case of micafungin against the type strain. It is notable that 22 of 60 (36.7%) anidulafungin plus nikkomycin Z and 24 of 60 (40%) micafungin plus nikkomycin Z combinations resulted in at least 100-fold decreases in CFUs (synergistic interaction) against wild-type isolates. Moreover, these combinations frequently resulted in fungicidal activity when nikkomycin Z was combined with anidulafungin (25/60 = 41.7%) or micafungin (12/60 = 20%) (Table 2 and Table 3). Notably, an antagonistic interaction was never observed. Nikkomycin Z significantly increased the killing activities of the two echinocandins against the isolate with an R1354H mutation in hot-spot 2 of *FKS1*, sometimes with synergism. Even against one of the two isolates with the established mutations in hot-spot 1 of *FKS1* (isolate 208), the 32 mg/L anidulafungin plus 8 mg/L nikkomycin Z produced CFU decreases without regrowth (Supplementary Materials Figure S2).

Our results are consistent with previous observations on the South African clade that echinocandins are weakly fungistatic [21,26]. Unfortunately, data on the in vitro pharmacodynamics of echinocandins against other clades are still limited. Nikkomycin Z MIC values for isolates of the South Asian and South American clades were similar to each other and lower than those observed with isolates from the East Asian clade. For isolates of the South African clade, the MIC values were lower than those reported for 100 isolates tested by Bentz et al. [16]. The lack of time–kill studies with nikkomycin Z alone or with echinocandins against different *C. auris* clades in that study precluded a comparison with our results.

Our results confirmed the positive interaction between echinocandins and nikkomycin Z using a time–kill methodology [19,27,28]. Poester et al. combined micafungin or caspofungin with nikkomycin Z (nikkomycin Z MICs were higher than 64 mg/L for all isolates) against 11 *C. auris* isolates using the checkerboard method. Micafungin with nikkomycin Z showed additive and synergistic interactions with six of seven and one of seven isolates (all belonging to the South Asian clade), respectively. The combination of caspofungin with nikkomycin Z proved to be synergistic against all five tested isolates of the South Asian clade and against the one tested isolate of the South African clade. However, killing studies were again not performed, and echinocandin resistant isolates were not tested [27].

An important strength of our study is that whole genome sequencing was performed with all isolates used in this study and included not only wild-type isolates but isolates with known mutations in hot-spots 1 and 2 of *FKS1*. One possible limitation is that the number of the isolates tested per some clades was relatively low and nikkomycin Z was tested only at 8 mg/L. However, in the killing studies, nikkomycin Z was tested with both echinocandins at five different concentrations (75 combinations of nikkomycin Z with each of anidulafungin and micafungin). Moreover, the time–kill methodology, in contrast to the checkerboard method, is suitable for measuring the ≥99.9 CFU decreases (i.e., fungicidal endpoint) and for distinguishing this from a fungistatic effect. The next-generation echinocandin rezafungin was not available for this study, and we did not test the third approved echinocandin, caspofungin, which may be regarded as another limitation. However, caspofungin susceptibility testing is not recommended using broth microdilution methodologies because of the significant interlaboratory variability observed using broth-based methods [29].

Briano et al. reported a significant association between multisite *C. auris* (South Asian clade) colonization (skin, respiratory and/or urinary) and development of *C. auris* candidemia in intensive care units during the COVID-19 outbreak (Genoa, Northern Italy), with a 27% crude 30-day mortality rate [30]. Alarmingly, 7 of 27 patients developed late recurrent candidemia during echinocandin treatment, and the MICs of caspofungin (4 mg/L) and amphotericin B (2 mg/L) increased against these isolates (in one case each). Patients with isolates showing low MICs to echinocandins were treated again with echinocandins, while the patient with an isolate that exhibited a high MIC to caspofungin was treated with anidulafungin plus flucytosine. The mortality was 57% (four of seven), suggesting that the killing activity of echinocandins against *C. auris* was weak among those critically ill patients [30]. Other authors have also reported septic metastatic complications (i.e., spondylodiscitis, meningitis and endo- and pericarditis) during echinocandin treatment [6,31]. The poor eradication of the fungus from the bloodstream seems, thus, fully in accordance with the weak in vitro fungistatic activity of echinocandins against wild-type *C. auris* [21,26].

Higher daily echinocandin doses may increase the cure rate among patients with invasive candidiasis. Although larger daily echinocandin doses were well tolerated in clinical situations, a significantly better cure rate was not detected [32,33]. Combination therapy is another strategy to combat invasive fungal diseases, including *C. auris* infections. Amphotericin B and its lipid formulations, voriconazole, isavuconazole and even flucytosine, have been added to the echinocandins to increase the cure rate with invasive *C. auris* infections. However, the full clinical significance of combination therapy is still unknown [12,15,18,34].

The mechanism responsible for the weak in vitro killing activity of echinocandins against *C. auris* is not well understood but may be related to aggregate formation by *C. auris* cells, as demonstrated both in vitro and in vivo [20,22]. Isolates of the South Asian clade have been reported to possess a higher basal chitin content in the cell wall compared to *C. albicans*, *C. tropicalis* and *C. guilliermondii*, as well as a higher basal expression of *CHS2* genes [35,36]. Pezotti et al. reported larger amounts of α-1, 3-glucans but smaller amounts of chitin in isolates from the East Asian and South African clades compared to their *C. albicans* counterparts using Raman spectroscopic methods [37]. As echinocandins inhibit β-1, 3-glucan but not α-1, 3-glucan synthesis, the relatively lower amount of β-1, 3-glucan in *C. auris* might explain the weaker inhibition of *C. auris* growth compared to *C. albicans* and other *Candida* species. However, echinocandin-induced cell wall damage increases chitin synthase activity with the resulting increased amounts of chitin in the cell wall compensating for the decreased β-1, 3-glucan levels [19]. In the current study, the simultaneous inhibition of β-1, 3-glucan and chitin synthases significantly increased the killing rate against the four clades, with the exception of one isolate from the South African clade, suggesting that depleting one of the cell wall components is insufficient for killing, and depleting both seems to impair the viability significantly, which would explain the synergism observed between the two echinocandins and nikkomycin Z. Synergism was found in the case of an isolate with mutations in hot-spot 2 of *FKS1*, which also exhibited low echinocandin MICs. The lack of synergism between the two echinocandins and nikkomycin Z against the two isolates with prominent mutations in hot-spot 1 of *FKS1* can be explained by a high basal or inducible chitin content compared to the wild-type isolates [38]. However, 8 mg/L of nikkomycin Z significantly increased the killing activity of 32 mg/L anidulafungin in the case of isolate 208 (with an HS1 S639P mutation), which had a low anidulafungin MIC (0.5 mg/L). This suggests that different echinocandin resistance mutations may affect synergy differently.

Since chitin is not found in mammals, the effects of nikkomycin Z are highly selective for fungi. The side effects are minimal, as demonstrated in preclinical and multidose human safety phase I trials [16,17,18]. Though nikkomycin Z alone is ineffective to treat *Candida* infections, earlier works and this study have shown that nikkomycin Z in combination with echinocandins produced synergistic or additive interactions without antagonism against *Candida* species [19]. The coadministration of nikkomycin Z with echinocandins might improve the outcome of invasive *C. auris* infections, particularly in immunosuppressed patients, warranting more efforts towards the cost-effective production and commercialization of nikkomycin Z [17,18].

## 5. Conclusions

Nikkomycin Z alone has no measurable killing activity, while anidulafungin and micafungin showed weak or moderately fungistatic activity against wild-type *C. auris*. The killing activities of anidulafungin and micafungin in the presence of nikkomycin Z significantly increased against *C. auris* at clinically attainable concentrations without antagonism, with the exception of one isolate from the South African clade. Our results suggest that by adding nikkomycin Z to anidulafungin or micafungin, the combination may be sufficiently potent to successfully treat invasive *C. auris* infections with the wild-type *FKS* genotype at clinically attainable concentrations. Further studies are needed to verify the clinical efficacy of echinocandin plus nikkomycin Z combinations.

## Figures and Tables

**Table 1:** MIC values of anidulafungin (ANI), micafungin (MICA) and nikkomycin Z (NIK) in RPMI 1640 against *Candida auris* isolates and type strain. MICs were determined using the CLSI broth microdilution method.

Isolates Number	Clade	Body Site	FKS Mutation		MIC (mg/L)	
				ANI	MICA	NIK
20	South Asian	Wound swab	HS1 WT HS2 R1354H	0.25	0.25	2
27 (NCPF 8991)	South Asian	Pleural fluid	HS1 WT HS2 WT	0.12	0.25	>16
28 (NCPF 8992)	South Asian	Central line	HS1 S639Y HS2 WT	4	4	16
196	South Asian	Blood	HS1 WT HS2 WT	0.12	0.25	>16
208	South Asian	Screening swab	HS1 S639P HS2 WT	0.5	2	16
Type strain (NCPF 13029 = CBS 10913)	East Asian	External ear	HS1 WT HS2 WT	0.03	0.12	>16
12372 (CBS 12372)	East Asian	Blood	HS1 WT HS2 WT	0.03	0.03	>16
12373 (CBS 12373)	East Asian	Blood	HS1 WT HS2 WT	0.06	0.06	16
2 (NCPF 8977)	South African	CSF	HS1 WT HS2 WT	0.03	0.25	8
204	South African	Tracheostomy	HS1 WT HS2 WT	0.03	0.12	>16
206	South African	Blood	HS1 WT HS2 WT	0.06	0.25	4
I-24	South American (Israel)	Blood	HS1 WT HS2 WT	0.06	0.12	4
I-156	South American (Israel)	Blood	HS1 WT HS2 WT	0.06	0.12	4
13108 (CDC B-13108)	South American (Colombia)	Hospital environment	HS1 WT HS2 WT	0.06	0.12	4
16565 (CDC B-16565)	South American (Colombia)	Hospital environment	HS1 WT HS2 WT	0.015	0.06	8

**Table 2:** Maximum changes in log CFU/mL compared to the starting inoculum in time–kill studies at different anidulafungin (mg/L), and anidulafungin (mg/L) plus 8 mg/L nikkomycin Z concentrations (mg/L) for the four *Candida auris* clades. Data in bold and underlined indicate a synergistic (more than 2 log CFU decreases in CFU) and fungicidal effect (at least 3 log decreases in CFU), respectively.

Clade	Isolate Number	Maximum Log Decreases/Increases in CFU in Time–Killing Experiments at the Indicated Anidulafungin and Anidulafungin + Nikkomycin Z Concentrations									
		Anidulafungin (mg/L)					Anidulafungin (mg/L) + Nikkomycin Z (mg/L)				
		0.25	1	8	16	32	0.25 + 8	1 + 8	8 + 8	16 + 8	32 + 8
South Asian	20	−0.27 *	−0.22 *	−0.29 *	−0.88	−0.74	−0.69	−1.0	−1.57	−1.48	−1.57
	27	−0.61	−1.04	−1.4	−1.32	−1.4	−2.3	−2.78	−3.16	−3.22	−3.05
	28	+2.39	−0.22 *	−0.19 *	+0.88	+0.54	+1.42	−0.05 *	−0.10 *	−0.10 *	−0.08 *
	196	−0.39	−0.57	−0.42	−0.62	−0.96	**−2.54**	**−3.02**	**−3.02**	**−3.02**	**−3.02**
	208	−0.08 *	+1.24	+1.22	+0.82	+0.52	+1.40	+1.10	−0.15 *	−0.10 *	−0.43
East Asian	Type strain	−1.15 *	−1.54 *	−1.98	−1.98	−3.0	−1.0	−1.13	−2.13	**−3.9**	**−3.9**
	12372	−0.58 *	−0.54 *	−1.42 *	−1.18 *	−1.23 *	−0.87 *	**−1.92**	**−3.85**	**−3.85**	**−3.85**
	12373	−0.52 *	−0.62 *	−0.62 *	−0.70 *	−0.80 *	−1.0	**−2.22**	**−4.0**	**−4.0**	**−4.0**
South African	2	+0.45	+0.24	+0.26	+0.32	+0.26	−1.28	−1.30	−1.41	−1.48	−1.60
	204	−0.35 *	−0.03 *	−0.19 *	+0.33	−0.15 *	−0.83	−0.95	−1.20	−1.32	**−1.82**
	206	+0.48	+0.51	+0.54	−0.19 *	−0.92 *	−0.34 *	−0.21 *	−0.22 *	−0.29 *	−0.44 *
South American (from Israel)	I-24	−0.33 *	−0.27 *	−0.22 *	−0.19 *	−0.11 *	−0.57	−0.69	−0.77	−0.82	−1.32
	I-156	−0.35 *	−0.18 *	−0–14 *	−0.22 *	−0–15 *	−0.85	−1.22	−1.41	−1.71	**−2.26**
South American (from Colombia)	13108	−0.96	−0.77	−0.78	−0.78	−0.48	**−4.08**	**−4.08**	**−4.08**	**−4.08**	**−4.08**
	16565	−2.3	−1.7	−1.09	−0.79	−0.99	**−4.15**	**−4.15**	**−4.15**	**−4.15**	**−4.15**

* Regrowth.

**Table 3:** Maximum changes in log CFU/mL compared to the starting inoculum in time–kill studies at different micafungin (mg/L), and micafungin (mg/L) plus 8 mg/L nikkomycin Z concentrations (mg/L) for the four *Candida auris* clades. Data in bold and underlined indicate a synergistic (more than 2 log CFU decreases in CFU) and fungicidal effect (at least 3 log decreases in CFU), respectively.

Clade	Isolate Number	Maximum Log Decreases/Increases in CFU in Time–Killing Experiments at the Indicated Micafungin and Micafungin + Nikkomycin Z Concentrations									
		Micafungin (mg/L)				Micafungin (mg/L) +Nikkomycin Z (mg/L)					
		0.25	1	8	16	32	0.25 + 8	1 + 8	8 + 8	16 + 8	32 + 8
South Asian	20	+1.02	−0.47 *	−0.35 *	−0.52 *	−0.44 *	**−1.78**	**−1.68**	−1.13	−0.95	−1.38 *
	27	−1.68	−2.51	−1.43	−1.27	−1.30	−2.38	−2.40	−2.51	−2.84	**−3.6**
	28	+1.45	+1.34	−0.83 *	−0.35 *	−0.44 *	+1.52	−0.16 *	−0.70 *	−0.57 *	−0.52 *
	196	−0.51	−0.69	−0.32	−0.24	−0.24	−2.32	−2.15	**−2.62**	**−2.62**	**−2.72**
	208	+1.67	+1.89	−0.08 *	−0.14 *	+1.13	+1.72	+1.52	−0–04 *	−0.04 *	−0.13 *
East Asian	Type strain	−0.67 *	−0.60	−0.56	−0.49 *	−0.51 *	−0.69	−0.64	−0.64	−0.54	−0.62
	12372	−0.54 *	−0.67 *	−0.54 *	−0.64 *	−0.72 *	**−1.51**	**−1.90**	**−2.12**	**−2.54**	**−3.15**
	12373	−0.30 *	−0.39 *	−0.52 *	−0.33 *	−0.39 *	−1.47	−1.49	**−1.70**	**−1.74**	**−2.64**
South African	2	+0.37	+0.34	+0.26	+0.36	+0.21	−1.46	−1.50	−1.60	**−1.67**	**−2.48**
	204	−0.08 *	−0.11 *	−0.05 *	−0.05 *	−0.11 *	−0.56	−0.65	−0.65	−0.78	−0.88
	206	+0.54	+0.51	+0.60	+0.48	+0.48	−0.23 *	−0.23 *	−0.23 *	−0.16 *	−0.22 *
South American (from Israel)	I−24	−0.30 *	−0.24 *	−0.19 *	−0.15 *	−0.24 *	−0.65	−0.83	−0.69 *	−0.83	−0.79
	I−156	−0.18 *	−0.30 *	−0.35 *	−0.35 *	−0.30 *	−1.30	−1.52	−1.49	−1.48	−1.42
South American (from Colombia)	13108	−0.86 *	−0.86 *	−0.95 *	−1.08 *	−1.08	**−3.13**	**−3.35**	**−4.26**	**−4.26**	**−4.26**
	16565	−1.15	−1.15	−0.79 *	−0.59 *	−0.77 *	**−4.26**	**−4.26**	**−4.26**	**−4.26**	**−4.26**

* Regrowth.

## Data Availability

The data shown and discussed in this paper have been deposited in the NCBI GenBank with the following BioProject no.: PRJNA865124.

## Supplementary Materials

The following supporting information can be downloaded at: https://www.mdpi.com/article/10.3390/pharmaceutics15051365/s1, Figure S1: Time-kill curves of nikkomycin Z in RPMI-1640 against isolates 20 (South Asian clade) and I-156 (bloodstream isolate from Israel, South American clade; Figure S2: Time-kill plots of anidulafungin, and anidulafungin plus nikkomycin Z in RPMI-1640 against isolates 28 and 208; Table S1: Time (hours) to reach 99.9% growth reduction (T99.9 = 3/*k*) from the starting inocula at different anidulafungin and anidulafungin plus 8 mg/L nikkomycin Z concentrations in RPMI-1640 against 4 Candida auris clades. Table S2: Time (hours) to reach 99.9% growth reduction (T99.9 = 3/*k*) from the starting inocula at different micafungin and micafungin plus 8 mg/L nikkomycin Z concentrations in RPMI-1640 against 4 Candida auris clades.
