# Supplementary material for: In Vitro Killing Activities of Anidulafungin and Micafungin with and without Nikkomycin Z against Four Candida auris Clades
Source: Pharmaceutics. 2023 Apr 29;15(5):1365. doi: 10.3390/pharmaceutics15051365 (PMC10222763; doi:10.3390/pharmaceutics15051365)
Supplement: Supplementary file 1 [file pharmaceutics-15-01365-s001.zip › Supplemental Figure S1.pptx]

## Slide 1
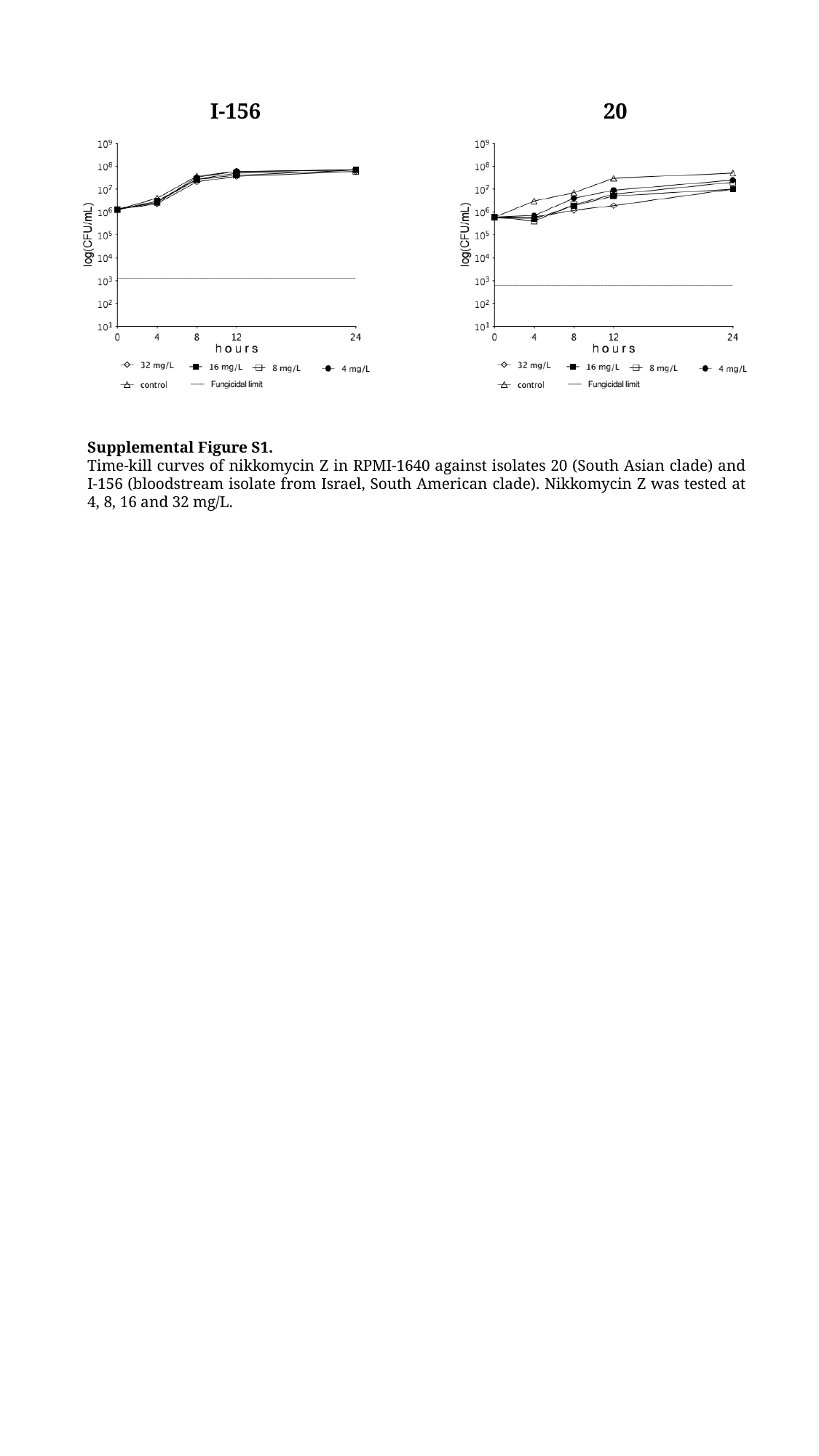

I-156
20
Supplemental Figure S1.
Time-kill curves of nikkomycin Z in RPMI-1640 against isolates 20 (South Asian clade) and I-156 (bloodstream isolate from Israel, South American clade). Nikkomycin Z was tested at 4, 8, 16 and 32 mg/L.
