# Supplementary material for: In Vitro Killing Activities of Anidulafungin and Micafungin with and without Nikkomycin Z against Four Candida auris Clades
Source: Pharmaceutics. 2023 Apr 29;15(5):1365. doi: 10.3390/pharmaceutics15051365 (PMC10222763; doi:10.3390/pharmaceutics15051365)
Supplement: Supplementary file 1 [file pharmaceutics-15-01365-s001.zip › Supplemental Figure S2.pptx]

## Slide 1
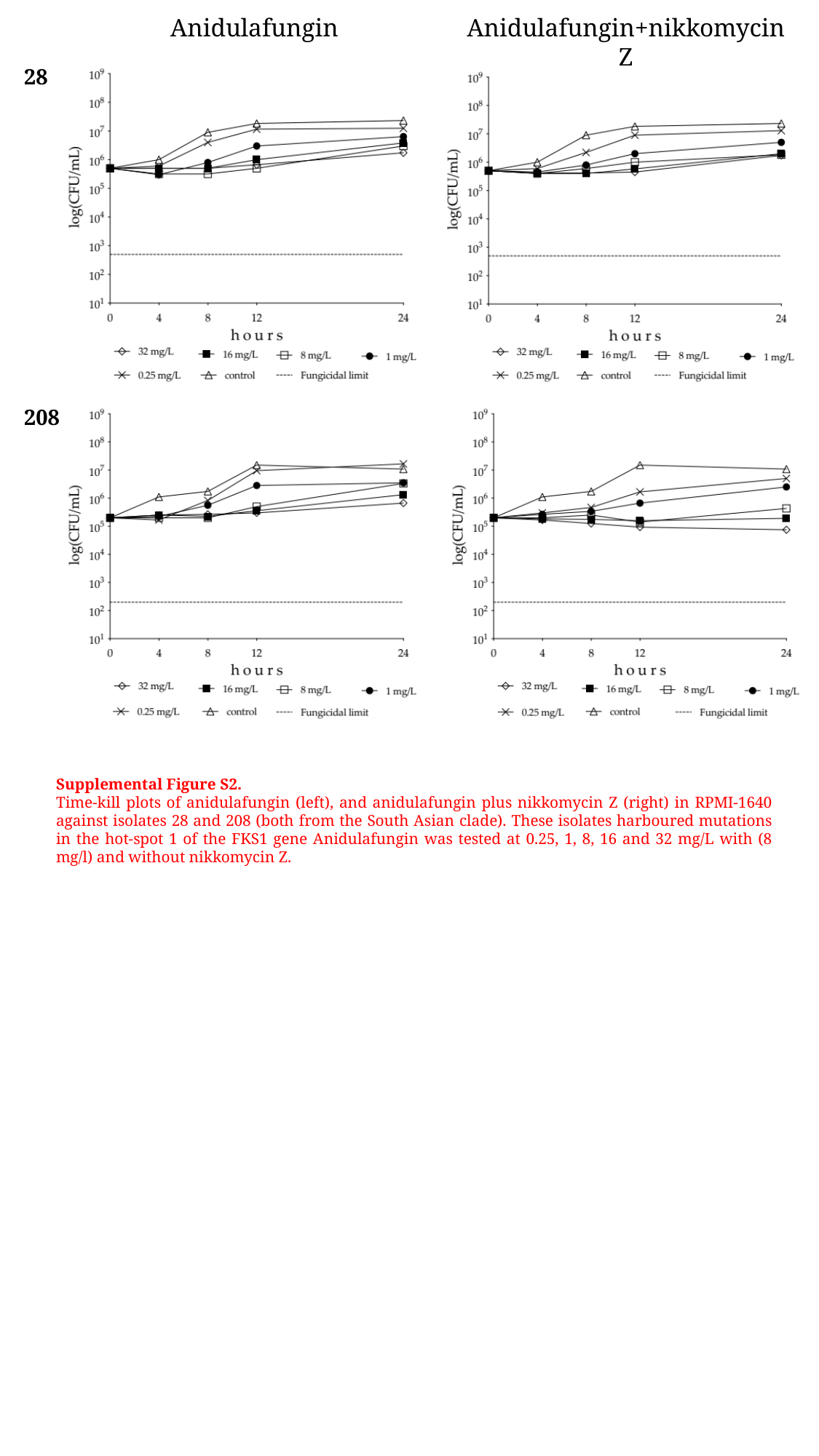

Anidulafungin
Anidulafungin+nikkomycin Z
28
208
Supplemental Figure S2.
Time-kill plots of anidulafungin (left), and anidulafungin plus nikkomycin Z (right) in RPMI-1640 against isolates 28 and 208 (both from the South Asian clade). These isolates harboured mutations in the hot-spot 1 of the FKS1 gene Anidulafungin was tested at 0.25, 1, 8, 16 and 32 mg/L with (8 mg/l) and without nikkomycin Z.
