# Supplementary material for: In Vitro Killing Activities of Anidulafungin and Micafungin with and without Nikkomycin Z against Four Candida auris Clades
Source: Pharmaceutics. 2023 Apr 29;15(5):1365. doi: 10.3390/pharmaceutics15051365 (PMC10222763; doi:10.3390/pharmaceutics15051365)
Supplement: Supplementary file 1 [file pharmaceutics-15-01365-s001.zip › Supplemental Table S1.pdf]

Supplemental Table S1. Time (hours) to reach 99.9% growth reduction ($T_{99.9}=3/k$) from the starting inocula at different anidulafungin and anidulafungin plus 8 mg/L nikkomycin Z concentrations in RPMI-1640 against 4 *Candida auris* clades. Measurable growth reduction times are shaded.

Type strain: NCPF 13029=CBS 10913. GR: growth occurred. NA: 99.9% growth inhibition not achieved.

**Clade**	**Isolate number**	$T_{99.9}$									
		Anidulafungin (mg/L)					Anidulafungin (mg/L)+nikkomycin Z (mg/L)				
		**0.25**	**1**	**8**	**16**	**32**	**0.25+8**	**1+8**	**8+8**	**16+8**	**32+8**
South Asian	20	GR	GR	NA	NA	NA	NA	NA	NA	NA	NA
	27	GR	NA	NA	NA	NA	NA	NA	5.8	3.9	2.6
	28	GR	GR	GR	GR	GR	GR	GR	GR	GR	GR
	196	NA	NA	NA	NA	NA	NA	8.7	6.5	5.3	4.7
	208	GR	GR	GR	GR	GR	GR	GR	GR	GR	NA
East Asian	Type strain	NA	NA	NA	NA	3.04	NA	NA	NA	2.9	1.7
	12372	NA	NA	NA	NA	NA	NA	NA	4.1	4.1	2.9
	12373	NA	NA	NA	NA	NA	NA	NA	4.1	2.6	2.7
South African	2	GR	GR	GR	GR	GR	NA	NA	NA	NA	NA
	204	GR	GR	GR	GR	GR	NA	NA	NA	NA	NA
	206	GR	GR	GR	GR	GR	GR	GR	GR	GR	GR
South American (from Israel)	I-24	GR	GR	NA	NA	NA	GR	GR	NA	NA	NA
	I-156	GR	GR	GR	GR	GR	NA	NA	NA	NA	NA
South American (from Colombia)	13108	NA	NA	NA	NA	NA	9.7	7.7	5.2	5.4	4.0
	16565	NA	NA	NA	NA	NA	6.7	8.3	5.6	3.95	4.6
