# Supplementary material for: In Vitro Killing Activities of Anidulafungin and Micafungin with and without Nikkomycin Z against Four Candida auris Clades
Source: Pharmaceutics. 2023 Apr 29;15(5):1365. doi: 10.3390/pharmaceutics15051365 (PMC10222763; doi:10.3390/pharmaceutics15051365)
Supplement: Supplementary file 1 [file pharmaceutics-15-01365-s001.zip › Supplemental Table S2.pdf]

Supplemental Table S2. Time (hours) to reach 99.9% growth reduction ($T_{99.9}=3/k$) from the starting inocula at different micafungin and micafungin plus 8 mg/L nikkomycin Z concentrations in RPMI-1640 against 4 *Candida auris* clades. Measurable growth reduction times are shaded.

Type strain: NCPF 13029=CBS 10913. GR: growth occurred. NA: 99.9% growth inhibition not achieved;

Clade	Isolate number	$T_{99.9}$ (hours)									
		Micafungin (mg/L)					Micafungin (mg/L) +nikkomycin Z (mg/L)				
		**0.25**	**1**	**8**	**16**	**32**	**0.25+8**	**1+8**	**8+8**	**16+8**	**32+8**
South Asian	20	GR	GR	GR	GR	GR	NA	NA	NA	NA	NA
	27	NA	NA	NA	NA	NA	NA	NA	NA	NA	18.1
	28	GR	GR	GR	GR	GR	GR	GR	GR	GR	GR
	196	NA	NA	NA	NA	NA	NA	NA	NA	NA	NA
	208	GR	GR	GR	GR	GR	GR	GR	GR	GR	GR
East Asian	Type strain	NA	NA	NA	NA	NA	NA	NA	NA	NA	NA
	12372	NA	NA	NA	NA	NA	NA	NA	NA	NA	23.7
	12373	NA	NA	NA	NA	NA	NA	NA	NA	NA	NA
South African	2	GR	GR	GR	GR	GR	NA	NA	NA	NA	NA
	204	GR	GR	GR	GR	GR	NA	NA	NA	NA	NA
	206	GR	GR	GR	GR	GR	GR	GR	NA	GR	GR
South American (from Israel)	I-24	GR	GR	GR	GR	GR	NA	NA	NA	NA	NA
	I-156	GR	GR	GR	GR	GR	NA	NA	NA	NA	NA
South American (from Colombia)	13108	NA	NA	NA	NA	NA	11.0	14.5	12.4	13.5	12.2
	16565	NA	NA	NA	NA	NA	11.2	12.3	17.4	12.5	19.1
